# RF Signal-Based UAV Detection and Mode Classification: A Joint Feature Engineering Generator and Multi-Channel Deep Neural Network Approach

**DOI:** 10.3390/e23121678

**Published:** 2021-12-14

**Authors:** Shubo Yang, Yang Luo, Wang Miao, Changhao Ge, Wenjian Sun, Chunbo Luo

**Affiliations:** 1 Glasgow College, University of Electronic Science and Technology of China, Chengdu 611731, China; 2018190607005@std.uestc.edu.cn; 2 Yangtze Delta Region Institute (Huzhou), University of Electronic Science and Technology of China, Huzhou 313001, China; 3 School of Information and Communication Engineering, University of Electronic Science and Technology of China, Chengdu 611731, China; sunwenjian@std.uestc.edu.cn; 4 Department of Computer Science, University of Exeter, Exeter EX4 4QF, UK; wang.miao@exeter.ac.uk; 5 James Watt School of Engineeering, University of Glasgow, Glasgow G12 8QQ, UK; 2357902G@student.gla.ac.uk

**Keywords:** unmanned aerial vehicles, UAV detection, UAV mode classification, Feature Engineering Generator, multi-channel deep neural network

## Abstract

With the proliferation of Unmanned Aerial Vehicles (UAVs) to provide diverse critical services, such as surveillance, disaster management, and medicine delivery, the accurate detection of these small devices and the efficient classification of their flight modes are of paramount importance to guarantee their safe operation in our sky. Among the existing approaches, Radio Frequency (RF) based methods are less affected by complex environmental factors. The similarities between UAV RF signals and the diversity of frequency components make accurate detection and classification a particularly difficult task. To bridge this gap, we propose a joint Feature Engineering Generator (FEG) and Multi-Channel Deep Neural Network (MC-DNN) approach. Specifically, in FEG, data truncation and normalization separate different frequency components, the moving average filter reduces the outliers in the RF signal, and the concatenation fully exploits the details of the dataset. In addition, the multi-channel input in MC-DNN separates multiple frequency components and reduces the interference between them. A novel dataset that contains ten categories of RF signals from three types of UAVs is used to verify the effectiveness. Experiments show that the proposed method outperforms the state-of-the-art UAV detection and classification approaches in terms of 98.4% and F1 score of 98.3%.

## 1. Introduction

Unmanned aerial vehicles (UAVs), also called drones, are gaining increasing popularity since they have high flexibility, ease of affordability, and exceptional capability. The recent advances in UAV technology have led to the proliferation of aerial services in our sky, e.g., emergency networks [1], healthcare system [2], surveillance system [3], coastal engineering [4], transportation engineering [5], assistance of distressed people [6], and many more [7]. Meanwhile, UAVs are employed to improve wireless communication, because they can provide connections between devices [8], increase the energy efficiency through trajectory optimization [9], assist resource allocation [10], and set up relay links [11]. Apart from single UAV’s usage, there is extensive research in applications of multiple UAVs, such as in radio navigation aids [12] and cellular networks [13]. The problems in communication networks between multiple UAVs before realizations of stable and reliable context-specific networks are overviewed [14].

Since the UAVs have flexibility, ease of affordability, and controllability, they may be utilized for malicious purposes and thereby pose potential security and privacy threats [15,16]. The threats contain eavesdropping, invading restricted regions, attacks on infrastructure, and colliding with people after losing control. To address these issues, efficient air traffic management becomes indispensable to ensure the safety of UAV flight and management [17], which is critical to both themselves and the flying environment, and property managers need to be aware of an approaching UAV. Hence, as the foundation for the following-up regulating measures, the methods for UAV detection and mode classification are urgently required.

Conventional detection methods, e.g., radars, acoustics, and vision, are constrained under some conditions. For example, the radar-based detection methods are restricted by the UAV sizes, the image-based methods have limits of distinguishing birds from drones, and acoustics-based methods are usually influenced by noise and have short detection distance [18]. Different from the conventional methods, RF signals can be detected at long distances and are less influenced by environmental factors. There is intensive research into classifying UAV types by RF signals. The work in [19] first detected the UAV signals, and then used neighborhood component analysis (NCA) and machine learning classifiers for classification of 15 UAV controllers types. Moreover, based on a Native Bayes approach and features of energy transient signal inputted into machine learning algorithms, 14 types of UAV RF signals are classified [20]. Bhattacherjee et al. in [21] utilized a keysight sensor to detect the UAV type by comparing the received RF signature with other UAVs’ RF signatures in a database. However, most existing works focus on detecting UAV types instead of UAV flight modes, which contain the information of UAVs’ operation status and are significant for UAV safety management. Therefore, in this paper, we proposed an effective RF signal-based method to not only detect UAVs’ types but also classify their flight modes.

The challenges of RF signal-based approaches are the similarities contained in the signals and the features of different frequency components. To tackle the challenges, our method first uses a Feature Engineering Generator (FEG) to extract features from RF signals. With the data preprocessed by FEG, we further design a DNN and a multi-channel deep neural network (MC-DNN) to classify the flight modes of UAVs. The multi-channel design separates different frequency components and reduces their corresponding interferences. The effectiveness of the method is verified based on a practical dataset in [22], where up to 10 categories of RF signals are included. The experiment results show that the proposed approach achieves an accuracy of 98.4% and an F1 score of 98.3%, and outperforms other state-of-the-art methods [22,23,24]. The main contributions of our work are summarized as follows.

- We design a joint FEG and MC-DNN approach for UAV detection and mode classification. The RF signals are preprocessed by FEG and then input into an MC-DNN for classification.
- In FEG, data truncation and normalization separates different components, the moving average filter removes the noise in the signals, and the concatenation exploits comprehensive details of the RF samples.
- We design MC-DNN to classify the signals preprocessed by the proposed FEG. The multi-channel input separates different frequency components of data to reduce interferences, and MC-DNN learns the classification effectively.
- We verify the joint approach through extensive experiments on an open dataset consisting of ten RF signal categories from three types of UAVs. Our method achieves high accuracy and F1 score and outperforms other methods.

The rest of the paper is organized as follows. Section 2 summarizes the related work. Section 3 describes the system model and problems. Section 4 presents the RF preprocessing and DNN structure. Section 5 provides the experimental results of our method. Finally, Section 6 draws some conclusions.

## 2. Related Works

Radio detection and ranging (RADAR) uses electromagnetic waves to collect information of flying objectives, such as distance and angle [25]. Thus, radars as active sensors are adopted for UAV detection, tracking, and classification. Most radar systems are designed based on Doppler signatures [25,26] and phase-interferometry [27]. Moreover, the work in [28] classifies two scenarios by not only using the micro-Doppler signature but also the cyclostationarity signature of the UAV signals and pseudo-Doppler principle. Although some progress has been made in the area of radar-based UAV detection, the relatively small radar cross-sections make UAVs invisible to radars and barriers influence the propagation of radar signals.

Acoustic sensors are applied for UAV detection. Data mining techniques are used based on acoustics sampling data, where the Hidden Markov Model was applied to analyze the emitted sound of UAVs [29]. A low-cost acoustic array of dynamically placed microphones was adopted to locate far-field small UAVs using a delay-and-sum beamforming algorithm [30]. However, the acoustic-based methods are influenced by high background sound and limited by the operating distance.

There are also vision-based approaches for UAV detection. A vision system based on a standard RGB digital camera to track a known UAV and assist automatic landing was presented in [31]. A method for a UAV to detect and track a cooperative flying vehicle was proposed based on template matching and morphological filtering [32]. The work in [33] constructed a YOLOv3 object detector to extract features from images using computer vision and convolutional neural network (CNN). Although being a promising technology, vision-based methods are sensitive to blurring images and line of sight limitations, such as cloud and fog, making the methods challenging to be used in real-world scenarios.

Different from the abovementioned methods, methods based on RF signals can be applied in the real world more easily, being less constrained by UAV shapes and the uncertainties in the acquisition environment. Meanwhile, the UAV RF signals can be captured at a long distance and contain abundant information about the UAVs’ flight modes [22,34], which cannot be easily achieved by other methods.

Since RF signals usually have a large amount of data, machine learning methods can be used to classify the RF signals. The neural networks in machine learning adapt the complex matches between the inputs and outputs of systems and are applied in many areas, such as speech recognition [35,36], human pose estimation [37], and image classification [38,39]. Neural networks automatically choose factors of the input to learn rather than relying on features picked by humans, which allows the methods to learn features more comprehensively and without biases. Therefore, using neural networks to classify UAV RF signals attracts considerable research efforts. The authors in [40] used wavelet transform analytics to extract unique signatures from the transient and steady state of the RF signals. A pretrained CNN-based model (SqueezeNet) was used to distinguish UAVs from interference and identify UAV types. The work in [41] trained CNNs using RF time-series images and spectrograms to classify 15 different drone controllers. The CNN model based on spectrograms was further applied denoising mechanism and was tested under different Signal-to-noise ratio (SNR) levels. However, the aforementioned research focused on the identification of UAV types from existing noise and interference instead of the UAV flight mode classification. Therefore, combining the advantages of RF signals and neural networks, we proposed a joint FEG and MC-DNN approach to not only detect UAV presence but also classify the UAV flight modes. We reduce the similarities and exploit the characteristics contained in different frequency components. Our method achieves high classification accuracy and F1 score, and our method outperforms other methods.

## 3. System Model and Problems

In this section, the system model is first introduced including RF signal acquisition, noise and interference, and RF signals in the frequency domain. Then, the challenges in UAV classification are presented as the problems to be tackled.

### 3.1. System Model

**Figure 1:**
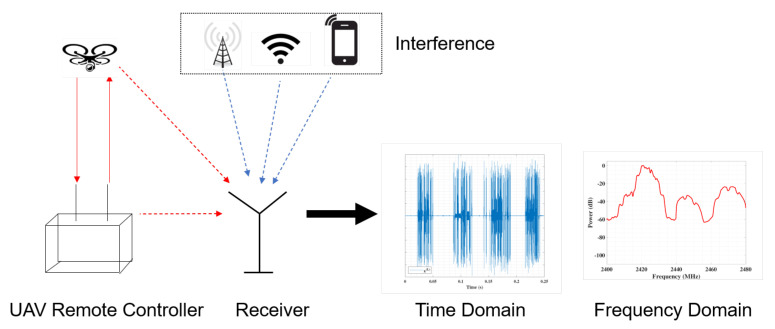
The system model of UAV RF signal acquisition.

We introduce the system model as in Figure 1 with a focus on the UAV operation signals’ characteristics, which serve as the basis for further proposed classification method.

#### 3.1.1. RF Signal Acquisition

There are mainly two kinds of RF signals generated between UAVs and their controllers: the uplink and the downlink. The uplink signal contains the controlling RF signal commands from the controller to UAVs, while the downlink one contains telemetry signals and video signals from UAVs to controllers. Most RF signals generated uniquely characterize UAVs due to the UAVs’ circuitry design and modulation techniques [19]. Besides, most UAVs are operated at frequencies around 2.4 GHz [42]. By passively and continuously listening to the communication between the UAVs and controllers, the unique RF signals for different types of UAVs with different flight modes can be collected. Herein, tools such as universal software radio peripheral (USRP) can be used for signal acquisition, and the sampling rates are set to be larger than the Nyquist rate to avoid aliasing.

Capturing signals of a larger bandwidth gives comprehensive information of different frequency components. However, the devices can have bandwidth constraints to capture the RF signals. Thus, the whole bandwidth is divided into low-frequency and high-frequency components, where each component is captured by respective device.

#### 3.1.2. Noise and Interference in RF Signals

The interference results from other wireless sources that are also operated in the same UAVs operating frequency band, such as Wi-Fi and Bluetooth. Besides, the signal to interference plus noise ratio (SINR) of the captured RF signals is related to the upper limit of classification. Thus, the captured signals with UAVs are modeled as the combination of UAV RF signals and the background signals with noise and interference, captured from the ambient environment without operating UAVs.

#### 3.1.3. RF Signals in Frequency Domain

The RF signals are typically captured in the time domain, but the signals in the frequency domain have latent characteristics. Besides, directly using time-domain signals for classification has some drawbacks: First, the time-domain signals usually have a large size of data, which requires high computation resources for preprocessing. The conversion to the frequency domain significantly reduces the data size. Second, if the time-domain signals are further divided into segments, the start and end of the segments are randomly chosen. This may result in a large portion of noise in some segments, and there are not enough features to conduct accurate UAV classifications. Third, many devices used to capture signals have bandwidth limits. The direct concatenation of bandwidth-limited time-domain signals is questionable while the frequency domain spectra are not. To avoid the abovementioned drawbacks and reveal more features, the RF signals are converted into the frequency domain by Fourier Transform.

### 3.2. Problems

The RF signal dataset includes the background RF signal and the RF signals of different UAVs with diverse flight modes. The UAV presence must be first classified. Consequently, the types of UAVs and flight modes of UAVs should be classified. The challenges lie in mainly four aspects: the similarities between distinct types of UAV signals, the similarities between the same type of UAVs with different flight modes, the existence of noise and interference, and distinguishing diverse features of different frequency components. This paper focuses on proposing a novel joint FEG and MC-DNN approach to overcome these challenges and classify the UAV flight modes accurately.

## 4. Methodology

To solve the problems, the proposed method consists of FEG preprocessing and MC-DNN. The method concentrates on boosting the discrepancies between each category of RF signals, separating different features represented by each frequency component, and learning representative features. Specifically, the FEG extracts more distinguishable features and reduces the influence of biases. The MC-DNN automatically selects features from preprocessed signals and learns the relationship between the input and the objectives effectively.

### 4.1. Feature Engineering Generator

The objective of feature engineering is to reveal features from raw data since the features represent the data better, the more accurate performance obtained. Thus, the FEG aims at separating different frequency components and reducing the similarities of signals. FEG uses three techniques: data truncation and normalization, moving average filter, and concatenation.

#### 4.1.1. Data Truncation and Normalization

The dataset of RF signal captured in the frequency domain is composed of low-frequency and high-frequency components. The components may own different features and exhibit different power levels. Thus, normalizing two components together leads to that the frequency component with small values is dominated by the other one. This means that the small values are normalized to nearly zero, and the value changes become almost invisible. To address this issue, we truncate the two components into two sub-datasets. Each sub-dataset is normalized individually to fully extract different features.

#### 4.1.2. Moving Average Filter

Since the existence of noise and the frequency spectra after Fourier Transform have oscillations, a *n*-point moving average filter is proposed to smooth the spectra and reduce the noise effects. While the noise is random, the UAV signals remain almost unchanged. The noise adds destructively in the filter, and the oscillations are reduced while keeping the substantial trend of UAV RF signals. The moving average filter is calculated as

(1) $$\bar{p}=\frac{1}{n}\sum_{i=0}^{n-1}p_{i}\;,$$

where $p_{i}$ is the input value, $\bar{p}$ is the output value, and *n* is the number of inputs. The output is the mean of adjacent *n* values. Due to different noise of the frequency components, the parameter *n* in Equation (1) for each component is chosen separately. When the frequency signals in the component have larger oscillations, bigger *n* is expected and more samples are averaged.

#### 4.1.3. Concatenation

Using individually normalized sub-dataset for classification can’t fully exploit the complete details of the dataset. Hence, the sub-datasets are concatenated to provide a comprehensive view of RF samples. Some concatenation ways alter the sub-datasets and reduce features, such as multiplying coefficients with the first samples of the high-frequency component [22]. Multiplication achieves continuity between the two components, but it results in diminishing small values and changing values. Direct concatenation connects the components without modification and keeps distinct respective features. Besides, the continuity between the low-frequency and high-frequency components is not necessary for classification.

In conclusion, the overall FEG algorithm is presented in Algorithm 1. The RF data is truncated into low-frequency and high-frequency components, resegmented, operated by Fourier Transform, moving average filtered separately, concatenated together, and labeled.
**Algorithm 1** Feature Engineering Generator Algorithm.**Require:**The original low-frequency time domain component *L*.The original high-frequency time domain component *H*.The number of samples in each data segment *M*.The number of categories of data from different types of UAVs with flight modes *N*.The points of moving average filter for the low-frequency and high-frequency components $n_{l}$ and $n_{h}$, respectively.**Ensure:** The Feature Engineering Generator preprocessed frequency domain data *D*.1:**for***n* in *N* **do**2:   Extract the time domain low-frequency component $L_{n}$ and high-frequency component $H_{n}$ of category *n*.3:   Resegment $L_{n}$, $H_{n}$ into new segments with *M* samples per segment $S_{L}$, $S_{H}$, respectively.4:   **for** *l* in $S_{L}$ **do**5:     Fourier transform *l*.6:     $n_{l}$-point moving average filter *l*.7:   **end for** *l*8:   **for** *h* in $S_{H}$ **do**9:     Fourier transform *h*.10:     $n_{h}$-point moving average filter *h*.11:   **end for** *h*12:   $S_{L}=\frac{S_{L}}{\max(S_{L})};S_{H}=\frac{S_{H}}{\max(S_{H})}$.13:   $S=(S_{L};S_{H})$.14:   $D_{n}=\left(S^{2};n\right)$.15:**end for***n*16:$D=(D_{1},D_{2},\cdots ,D_{n})$.

### 4.2. DNN Structure

Given the RF signals preprocessed by FEG, DNNs are designed to solve the multi-class classification problem. DNNs can automatically select and learn the features in the RF signals. A well-designed DNN is capable of adapting the relationship between the input and objective. In this section, a DNN structure is first designed for classification. The performance of the DNN is also contrasted as a baseline. Next, the multi-channel technique is applied and a multi-channel input DNN is designed for better classification performance.

#### 4.2.1. Deep Neural Network

**Figure 2:**
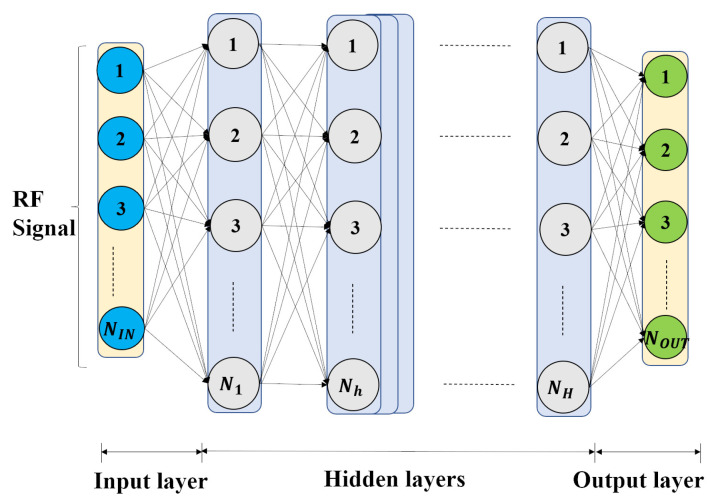
The structure of the DNN with an input layer, *H* hidden layers and an output layer.

Based on feedforward artificial neural networks called multilayer perceptron, a DNN in Figure 2 is designed to classify the RF signals, which includes the model input and output, DNN structure, and loss function.

##### Input and Objective

The DNN classifies the RF signal data, and the classes are encoded by one-hot encoding into sequences of numbers with 1 representing the corresponding class and 0 representing other classes. Each objective for one piece of input data is a vector, and its dimension is the number of classes.

##### Deep Neural Network Structure

The DNN has *H* hidden layers with $N_{h}$ neurons in layer *h*, and the structure details are explained in [43]. The leftmost layer is the input layer with $N_{IN}$ neurons, being equal to the dimension of input RF signals preprocessed by FEG. The rightmost layer is the output layer with $N_{OUT}$ neurons, being equal to the number of classes. Each layer receives all the outputs of the previous layer and operates the calculation as follows.

(2) $$\begin{array}{cc} z_{l} & =W_{l}^{T}a_{l-1}+b_{l}\;,\\ a_{l} & =\delta_{l}\left(z_{l}\right)\;, \end{array}$$

where $a_{l}$ is the output vector of layer *l*, $a_{l-1}$ is the output vector of the previous layer, $W_{l}$ is the weight vector, $b_{l}$ is the bias vector, and $\delta_{l}\left(\cdot \right)$ is an activation function, e.g., rectified linear unit function (ReLU) and Softmax function. The weights and biases of each layer are determined through a supervised learning process. A loss function is minimized by a gradient descent algorithm.

##### Loss Function

The DNN’s loss function *L* is defined as the mean square error between the outputs and the objectives as follows.

(3) $$L\left(d_{i},\hat{d_{i}}\right)=\frac{1}{C}\sum_{c=1}^{C}{\left(d_{i}\left(c\right)-\hat{d_{i}\left(c\right)}\right)}^{2}\;,$$

where $d_{i}$ is a vector of the objectives, $\hat{d_{i}}$ is a vector of the final layer outputs, and *C* is the total number of outputs. The objective of the DNN is to minimize the loss function. During this process, the DNN learns the relationship and improves accuracy.

##### Stratified K-Fold Cross-Validation

To estimate the performance and effectiveness of the DNN on a limited dataset, stratified K-fold cross-validation [44] is adopted. The signals and objectives are shuffled randomly and divided into *K* folds evenly. The number of samples per category in each fold is proportional to the category’s portion in the dataset. There are *K* training and testing cycles, where K−1 folds are the training set and the remaining fold is for testing. Each fold is used to test once and train K−1 times. Training on the same training set can lead to overfitting on the training set and perform badly on unseen data. The overall performance metrics are summarized by taking the mean of *K* results. The cross-validation average result provides a steady evaluation and objectively reflects the performance of a network.

##### Confusion Matrix

The confusion matrix, or error matrix, is used to evaluate the performance of a classifier [45] by giving details into the errors and their types. It visualizes the overall accuracy by comparing the actual objectives and predicted classes. The columns of the confusion matrix represent the output class, while the rows represent the predicted classes. Several performance metrics are specified in the confusion matrix, e.g., recall, precision, false discovery rate (FDR), false-negative rate (FNR), accuracy, error, and F1 score.

#### 4.2.2. Multi-Channel DNN

**Figure 3:**
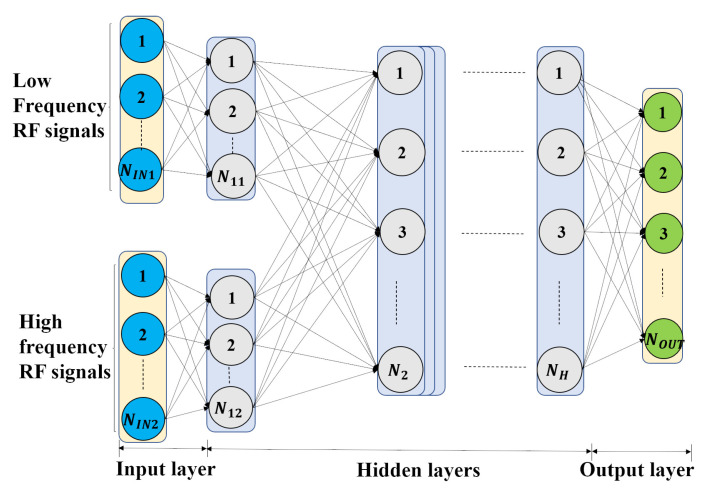
The structure of multi-channel deep neural network (MC-DNN).

Various factors contribute to the final classification result and the factors have little correlation with each other. Here, the low-frequency and high-frequency components have respective features and relationships to the objective. The multi-channel input technique enables the model to consider more possible factors and prevent factors from interfering with each other. Hence, based on DNN, multi-channel DNN (MC-DNN) is designed. Different from the first hidden layer following two components in DNN, the two FEG preprocessed components are input separately to the MC-DNN in Figure 3. The first channel input is the low-frequency component and the second channel input is the high-frequency component. The two channels are followed by the first hidden layer, with $N_{11}$ and $N_{12}$ neurons connected to two inputs, respectively. Afterwards, there are *H* hidden layers and an output layer. The MC-DNN isolates the two frequency components, and better learns the respective classification features for each component.

#### 4.2.3. Learning Rate Decay

Learning rate is a significant hyperparameter in training a DNN since it defines the step size the DNN parameters update every time. If the learning rate is set too large, the parameters can learn too fast and oscillate around the optimal loss function minimization point without converging. On the contrary, if too small, the parameters can learn too slowly and overfit the training data. Both situations affect the DNN classification performance. The best choice of defining the learning rate is to set it large at first and reduce it gradually. This means the DNN learns fast at first and slowly when approaching the optimal minimization point. Thus, the learning rate cosine decay technique [46] is adopted, where the learning rate decreases as follows.

(4) $$\eta_{t}=\frac{1}{2}\left(1+\cos\left(\frac{t\pi }{T}\right)\right)\eta \;,$$

where the total number of epochs is *T*, η is the initial learning rate, and $\eta_{t}$ is the learning rate at epoch *t*. The learning rate decreases from an initial value η to approximately 0 following the cosine function. The speed of cosine decay is slow at the beginning, linear in the middle, and slow again at the end. This training technique enables the MC-DNN to learn fast at first and converge to the loss function minimization point in the end.

## 5. Experiments

In this section, the dataset in [22] is used to verify the effectiveness of our method. First, the details of the dataset are introduced. Then, our method is applied step by step, and the performance is compared. The final result of our method is also contrasted with other methods.

We use the performance of DNN and data without preprocessing as the baseline. Every other step of FEG preprocessing is applied based on previous ones. For example, the second preprocessing technique introduced is the data truncation and normalization, so the truncated and normalized data input into DNN is the second experiment. After cumulatively applying the FEG steps, the preprocessed data is input into MC-DNN for experiments.

**Figure 4:**
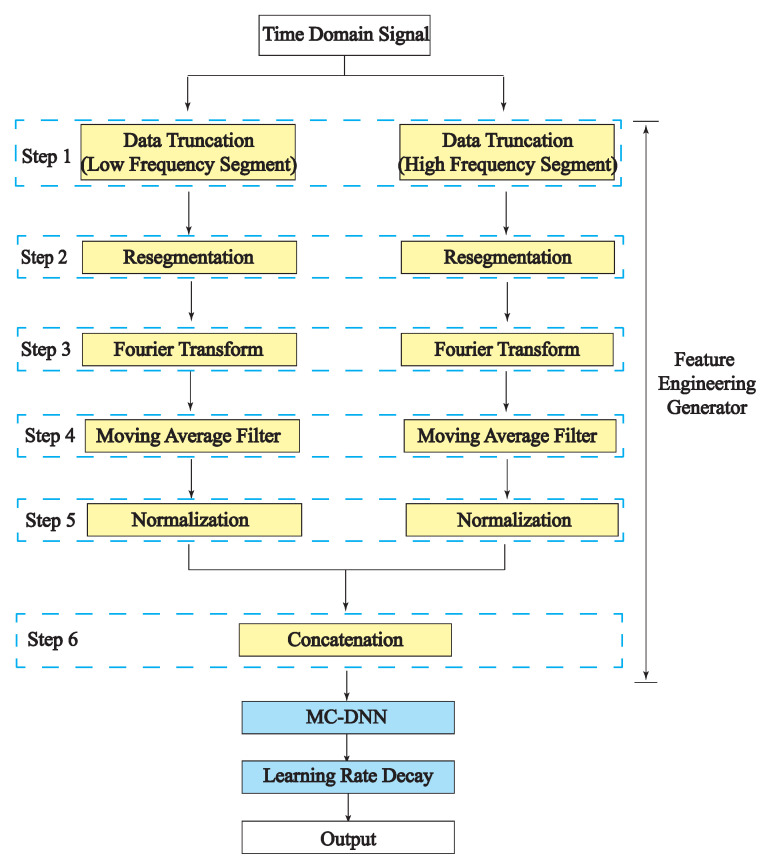
The overall flow chart of the proposed method including FEG and DNNs for classification. The RF signals are preprocessed by the steps of FEG, and then input into DNNs for classification.

The entire system model of the FEG and MC-DNN is presented in the flow chart in Figure 4. The FEG preprocessing steps in the flow chart implemented for each experiment are carefully labeled.

### 5.1. Dataset

**Figure 5:**
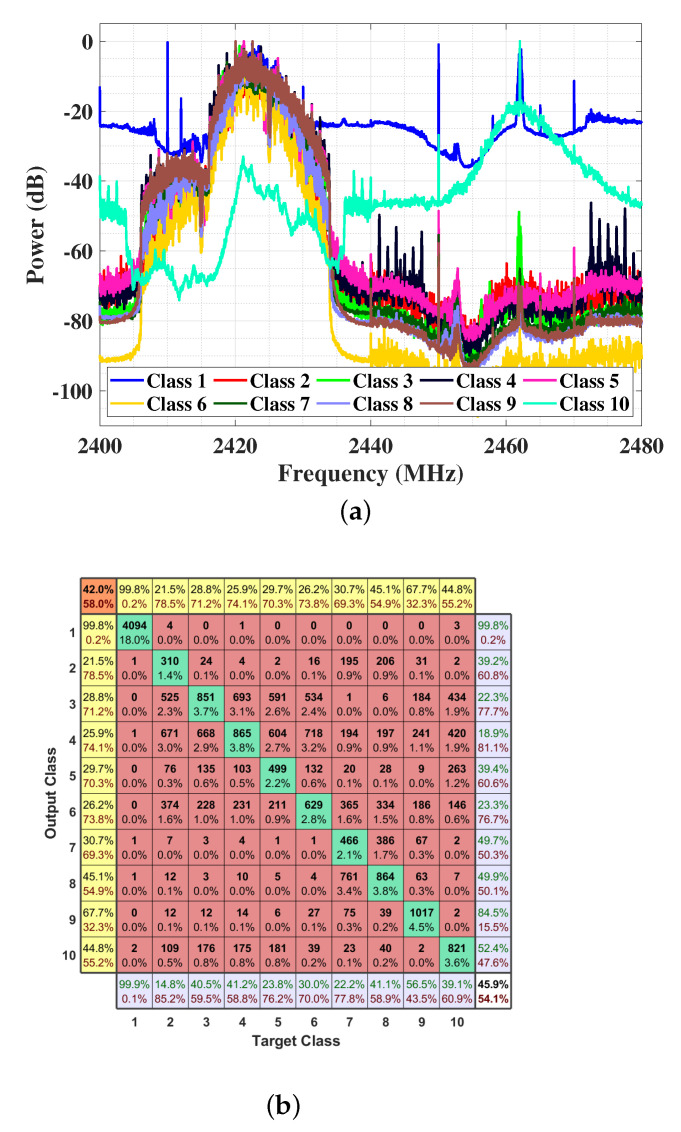
(**a**) The average spectra. (**b**) The confusion matrix. The spectra of the original RF signal dataset, which contains low-frequency component and high frequency component, and the confusion matrix of training DNN using the dataset. Class 1 is the background noise and class 2–10 are different flight modes of three kinds of UAVs.

To verify the effectiveness of our method, the dataset for UAV detection in [22] is adopted. It consists of data from three different types of UAVs: Parrot Bebop, Parrot AR Drone, and DJI Phantom 3. Each type of UAV has four flight modes: mode “On”, mode “Hovering”, mode “Flying without video recording”, and mode “Flying with video recording”. The dataset contains 10 categories of RF signals: background with no UAVs, four flight modes of UAV “Parrot Bebop”, four flight modes of UAV “Parrot AR Drone” and mode on of UAV “DJI Phantom 3”. Each category of data is collected by two RF receivers that intercept the UAV’s communications in each flight mode simultaneously. Because the RF receivers have bandwidth constraints, two receivers record low-frequency and high-frequency components, respectively. Then, the received time-domain data is labeled and stored subsequently. The originally captured data segments have $10^{7}$ samples per segment. Next, the segments are divided into smaller segments with $10^{5}$ samples to increase the amount of data for further supervised learning. The segments with $10^{5}$ samples are processed by Fourier Transform into the frequency domain. The high-frequency component and low-frequency component are then concatenated. To ensure the concatenation to be continuous, the first 10 samples of the high-frequency component are multiplied by a coefficient determined by the low-frequency component. The final dataset spectra is shown in Figure 5a.

### 5.2. DNN

The parameters for the DNN in Figure 5b are as follows. DNN has five layers in total, with one input layer, three hidden layers, and one output layer. Each hidden layer has 128 neurons and the output layer has 10 neurons, the same as the number of classes. The activation function for the output layer is the Softmax function, and the one for other layers is the ReLU function. The DNN is trained by an Adam optimizer, minimizing the mean square error loss function. The number of epochs is set to 300 and the batch size is 32. Note that the batch size is set to the powers of 2 to make calculations more efficient.

Using the dataset without signal FEG preprocessing as input to DNN, we get a baseline accuracy of 45.9% and an F1 score of 42.0%. The confusion matrix of the evaluation on the performance is shown in Figure 5b.

The details of confusion matrix plots are illustrated as follows (see Figure 5b for an example). Ten inner rows represent the output classes and ten inner columns represent the ten objective classes. The diagonal cells in green show the correct predicted samples and rate. Other cells in the inner rows and columns in red correspond to the number and portion of wrongly predicted samples. The top row and the leftmost column in yellow color demonstrate the F1 scores of ten class predictions in green font and the complementary of F1 score in red font. The top leftmost cell in orange averages all the F1 scores and the complementary ones. Besides, the purple bottom row illustrates the recall in green font and FNR in red font. The purple rightmost column presents the precision in green font and the FDR in red font. The bottom rightmost cell in white reveals the average accuracy in black and the complementary error rate in red. The precision, recall, and F1 score can be calculated as follows.

(5) $$\mathrm{precision}=\frac{\mathrm{TP}}{\mathrm{TP}+\mathrm{FP}}\;$$

(6) $$\mathrm{recall}=\frac{\mathrm{TP}}{\mathrm{TP}+\mathrm{FN}}\;$$

(7) $$F1\;\mathrm{score}=2\frac{\mathrm{precision}\times \mathrm{recall}}{\mathrm{precision}+\mathrm{recall}}=\frac{2\mathrm{TP}}{2\mathrm{TP}+\mathrm{FP}+\mathrm{FN}}\;$$

where TP means true positive, FP means false positive, and FN means false negative.

### 5.3. Joint DNN and Feature Engineering Generator

We evaluate each FEG preprocessing technique step by step cumulatively, and the data is input to the DNN for training.

#### 5.3.1. Data Truncation and Normalization

After applying data truncation and normalization, FEG preprocessing steps 1, 2, 3, and 5 in Figure 4 are implemented. The DNN is trained using the low-frequency component and high-frequency component separately, aiming at exploring more features contained in each component.

**Figure 6:**
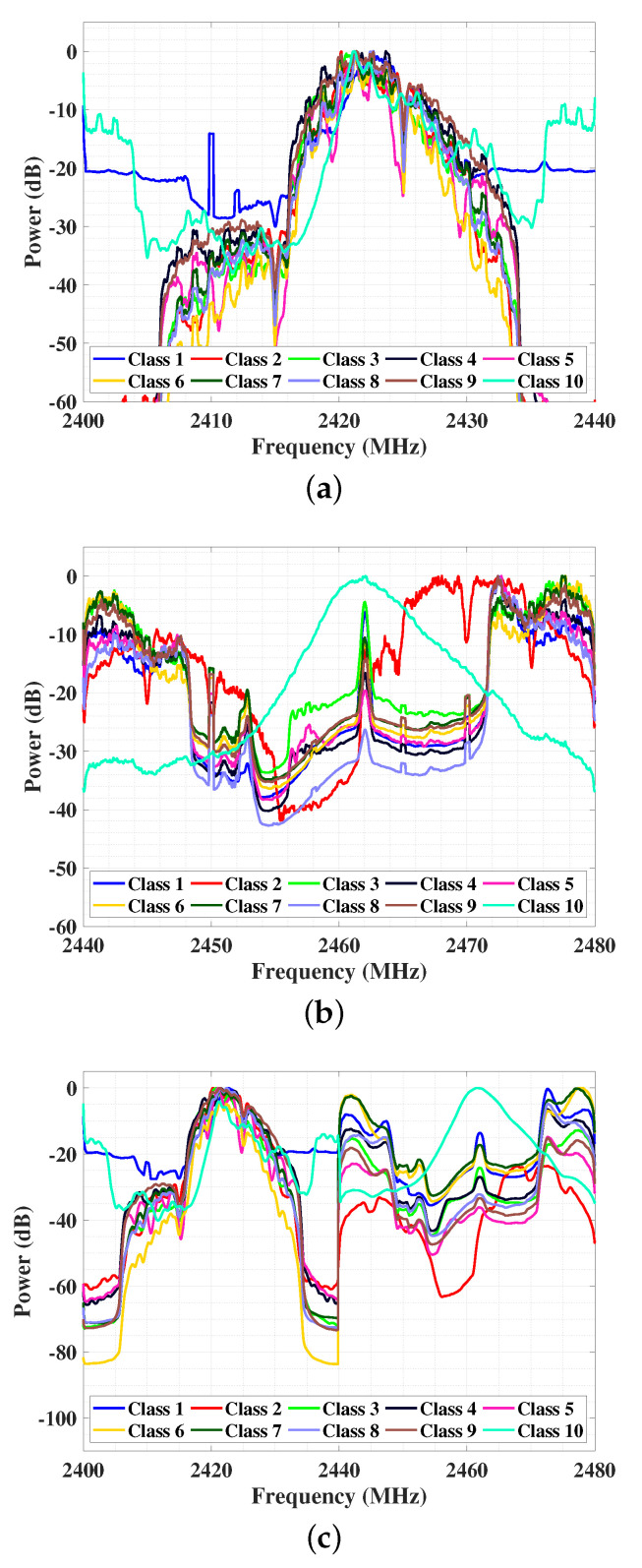
(**a**) Average spectra of the low-frequency component. Preprocessing steps 1, 2, 3, and 5 in the flow chart are implemented. (**b**) Average spectra of the high-frequency component. Preprocessing steps 1, 2, 3, and 5 in the flow chart are implemented. (**c**) Average spectra of the concatenation. Preprocessing steps 1-6 in the flow chart are implemented. The spectra of the low-frequency component, high-frequency component, and direct concatenation of 20-point moving average filtered low-frequency component and the 40-point moving average filtered high-frequency component. Class 1 is background noise and class 2–10 are different flight modes of three kinds of UAVs.

**Figure 7:**
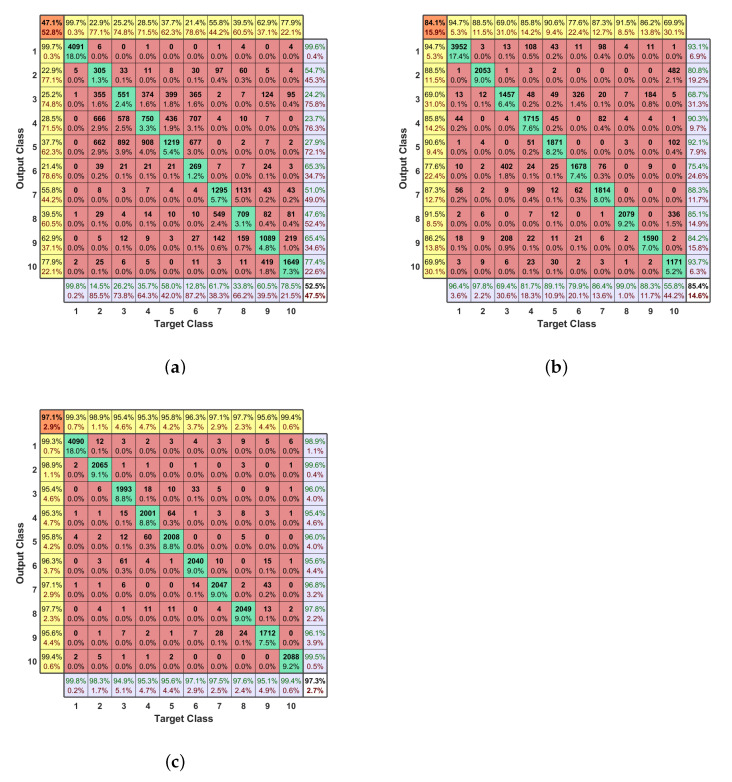
(**a**) The confusion matrix of the DNN with low-frequency component as input. (**b**) The confusion matrix of the DNN with high-frequency component as input. (**c**) The confusion matrix of the DNN with the concatenation as input. The confusion matrices of the DNN that take input of low-frequency component, high-frequency component, and direct concatenation of filtered and normalized components. Class 1 is background noise and class 2–10 are different flight modes of three kinds of UAVs.

The low-frequency component of the data, plotted in Figure 6a, is first truncated and normalized. Note that the plotted low-frequency spectra are processed by a 10-point average filter for visualization. As in the figure, the region of signals between approximately 2415 MHz and 2435 MHz have similar trends and peaks, which makes the classification difficult. Only the two regions near 2400 MHz and 2440 MHz have some visible differences. The low-frequency component of data is used to train the DNN, which achieves an accuracy of 52.5% and an F1 score of 47.1%. The confusion matrix of training DNN using the normalized low-frequency component is plotted in Figure 7a. The accuracies of some categories of flight modes are around 25%, which means the category is not classified correctly. The performance requires further improvements.

The high-frequency component in Figure 6b is preprocessed in the same way as the low-frequency one, i.e., truncated and normalized. Note that the high-frequency component spectra plot is also processed by a 10-point average filter. Improved accuracy of 85.4% and F1 score of 84.1% are achieved by training DNN using the high-frequency component. The great improvement is because the high-frequency component has fewer similarities and more distinct features between the ten categories of signals. Besides, the data truncation and normalization avoid the high-frequency component from being dominated by the low-frequency component. The confusion matrix of using the high-frequency component to train is plotted in Figure 7b.

#### 5.3.2. Moving Average Filter

In addition to the steps in Section 5.3.1, the performance of the moving average filter (step 4) is evaluated in this subsection. Specifically, the two components are processed by steps 1–5 and used to train DNN separately. Because the characteristics of each component of data are different, the parameter *n* is different. To find an optimal result for each component, a sequence of values for the moving average filter parameter *n* is tested. The accuracies and F1 scores of the DNN with filtered low-frequency and high-frequency components as inputs are illustrated in Table 1. Experiment results show that using moving average filters is effective on both components. The frequency signals have reduced noise and more distinct features after filtering. The low-frequency component achieves an accuracy of 65.5% and an F1 score of 62.2% after being preprocessed by a 20-point moving average filter. The 40-point moving average filtered high-frequency component achieves an accuracy of 90.6% and an F1 score of 89.7%.

#### 5.3.3. Concatenation

This subsection evaluates the effectiveness of concatenation (step 6) based on steps 1–5. All preprocessing steps 1–6 in FEG preprocessing are implemented. The low-frequency component filtered by the 20-point moving average filter and the high-frequency component filtered by the 40-point moving average filter are concatenated directly. The concatenated data is shown in Figure 6c, which has less information loss compared with the concatenation method in [22]. The accuracy of DNN trained with the concatenated data is 97.3% and the F1 score is 97.1%. The resulting confusion matrix is presented in Figure 7c. This proves that complete data details achieve better performance.

### 5.4. Joint MC-DNN and Feature Engineering Generator

The DNN is developed into the MC-DNN, and then the learning rate decay is added. The input in this section is the data fully processed by FEG.

#### 5.4.1. Multi-Channel Input

**Figure 8:**
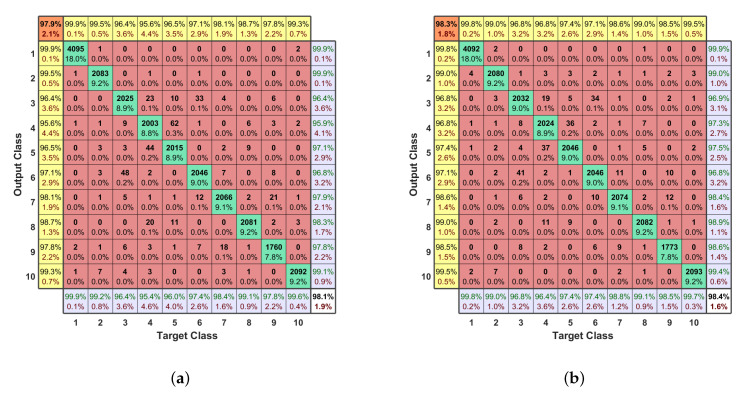
(**a**) The confusion matrix of the MC-DNN. (**b**) The confusion matrix of the MC-DNN with learning rate cosine decay. The confusion matrices of training MC-DNN and MC-DNN with learning rate decay using the fully FEG preprocessed data. Class 1 is background noise and class 2–10 are different flight modes of the three kinds of UAVs.

The designed MC-DNN in Figure 3 has double-channel inputs, the first hidden layer consisting of two parts for two inputs, three hidden layers, and one output layer. There are 256 neurons in the first hidden layer, and 128 neurons in other hidden layers. The result confusion matrix of using the preprocessed data to train MC-DNN is shown in Figure 8a, where the accuracy is improved to 98.1% and the F1 score is improved to 97.9%. This is because multi-channel input separates the inputs and makes the follow-up dense layers learn differently. Furthermore, two separate channels of inputs add more parameters in the MC-DNN, i.e., weights and biases. More parameters can better fit the complex relationships.

#### 5.4.2. Learning Rate Decay

Cosine learning rate decay allows the MC-DNN to converge faster and fit the data. The initial learning rate η in Equation (4) is set to 0.01. After using the fully FEG preprocessed data as input, the result confusion matrix is presented in Figure 8b. The learning rate cosine decay training technique increases the accuracy to 98.4% and the F1 score to 98.3%.

### 5.5. Comparison

The performance comparison between FEG techniques and DNN structures is presented in Table 2. As shown in Table 2, the accuracy and F1 score are achieved with each preprocessing technique and DNN structure are additionally applied. Eventually, our method achieves an accuracy of 98.4% and an F1 score of 98.3% for the classification. Meanwhile, the FEG plays an important role in advancing the performance, improving from 45.9% to 97.3%. The DNN structure improve from 97.3% to 98.4%. The baseline accuracy and F1 score of training DNN are only 45.9% and 42.0% since techniques are not applied to extract and learn the signal features. The improved accuracy and F1 score verify the effectiveness of our method.

Our method also outperforms other methods in terms of accuracy and F1 scores [22,23,24] applying on the same dataset. The work in [22] offers an open dataset and designed a three-hidden-layer DNN for classification with the frequency-domain data as input. The proposed method classifies the presence and UAV types with accuracies of 99.7% and 84.5%. However, the overall ten-class accuracy and F1 score obtained are 46.8% and 43.0%. This accuracy may only prove the feasibility and not support accurate flight mode classification. Furthermore, Convolutional Neural Networks (CNN) are designed based on this dataset [23]. Dropout layers are added to prevent overfitting problem. Two separate CNN structures with different hyper-parameters are proposed for UAV detection and flight mode identification. The CNN structure for classification contains 6 one-dimensional (1D) convolutional layers and dropout rate of 0.2. The proposed model derives an accuracy of 59.2% and an F1 score of 55.1% for the ten-class classification. The multi-channel 1D CNN in [24] includes a feature extractor and a classical MLP. The captured 80 MHz frequency spectrum is divided into 8 channels evenly, with each channel a separate input to the classifier. The multi-channel 1D CNN uses Adam optimizer and cross-entropy loss function. An accuracy of 87.4% and an F1 score of 77% are obtained by this model. The comparisons of accuracy and F1 score between our method and others are presented in Table 2.

Our method improves the accuracy and F1 score to 98.4% and 98.3%, respectively. Compared with other methods, our method first focuses on dataset preprocessing for full feature extraction. The preprocessing prepares the data for further MC-DNN learning. Moreover, the MC-DNN in our channel learns the low-frequency and high-frequency components separately, which contributes to the final performance. The additional learning rate decay is also effective for the convergence to good performance. Our method’s high accuracy significantly reduces potentially high errors in classifying UAV modes. This leads to practically applicable solutions in real-world scenarios. From the comparison with other methods, the effectiveness and great performance improvement of the method in this paper are significant.

## 6. Conclusions

We proposed a joint approach of FEG and MC-DNN to detect UAV presence and classify UAV flight modes. The challenges of RF classification mainly focus on the high similarities between categories of RF UAV signals and the different characteristics represented by frequency components of data. To address these challenges, our method first preprocessed the RF signals by FEG using data truncation and normalization, moving average filter, and concatenation. A carefully designed MC-DNN with learning rate cosine decay, modified based on DNN, was proposed to classify the preprocessed data. The experiments showed the effectiveness of our method, which classifies ten categories with an accuracy of 98.4% and F1 score of 98.3%, and outperforms the state-of-the-art solutions. The proposed method could be extended by other researches on UAV detection and classification performance improvement, including more effective feature extraction as well as novel classification models focusing on finer frequency details.

## Figures and Tables

**Table 1:** The accuracies and F1 scores of DNN with low-frequency data and high-frequency data processed by $n_{l}$ and $n_{h}$ point moving average filters as inputs, respectively. Preprocessing steps 1–5 in the flow chart are implemented.

Data		n-Point Moving Average Filter										
		0	5	10	15	20	25	30	35	40	45	50
Low-frequency component	accuracy (%)	52.5	58.7	58.0	61.1	**65.5**	64.1	60.8	64.1	61.7	63.3	62.9
	F1 score (%)	47.1	53.1	52.0	56.7	**62.2**	59.5	55.8	60.5	56.8	59.7	59.2
High-frequency component	accuracy (%)	85.4	84.2	82.7	89.0	87.9	87.2	89.8	89.6	**90.6**	89.5	90.4
	F1 score (%)	84.1	82.8	81.2	88.0	86.8	85.8	88.8	88.5	**89.7**	88.4	89.4

**Table 2:** The comparison of different combinations of FEG and DNN techniques and against existing methods. The FEG preprocessing techniques are implemented cumulatively, and the number represents the preprocessing steps in Figure 4. The operations corresponding to the steps are: step 1-data truncation, step 2-resegmentation, step 3-Fourier Transform, step 4-moving average filter, step 5-normalization, and step 6-concatenation. Since there are truncation and concatenation, the accuracies for low-frequency and high-frequency components are separately labeled in the brackets. The last three rows show the comparisons between our method and other methods.

Method	Accuracy		F1 score	
Unpreprocessed data + DNN	45.9%		42.0%	
Preprocessing steps 1,2,3,5 + DNN	52.5% (low)	85.4% (high)	47.1% (low)	84.1% (high)
Preprocessing steps 1–5 + DNN	65.5% (low)	90.6% (high)	62.2% (low)	89.7% (high)
Preprocessing steps 1–6 + DNN	97.3%		97.1%	
Preprocessing steps 1–6 + MC-DNN	98.1%		97.9%	
Preprocessing steps 1–6 + MC-DNN + Learning rate decay	**98.4%**		**98.3%**	
Classification method in [22]	46.8%		43.0%	
Classification method in [23]	59.2%		55.1%	
Classification method in [24]	87.4%		/	

## Data Availability

The dataset adopted is available online https://data.mendeley.com/datasets/f4c2b4n755/1 (accessed in June 2020).

## Abbreviations

The following abbreviations are used in this manuscript:

UAV	Unmanned Aerial Vehicle
RF	Radio Frequency
FEG	Feature Engineering Generator
DNN	Deep Neural Network
MC-DNN	Multi-Channel Deep Neural Network
CNN	Convolutional Neural Network
NCA	neighborhood component analysis
RADAR	Radio detection and ranging
SNR	Signal-to-noise Ratio
SINR	Signal to Interference plus Noise Ratio
USRP	Universal Software Radio Peripheral
ReLU	rectified linear Unit Function
1D	One Dimentional
FDR	False Discovery Rate
FNR	False-Negative Rate
